# Collision‐Induced Fragmentation of Oligosaccharides: Mechanistic Insights for Mass Spectrometry‐Based Glycomics

**DOI:** 10.1002/anie.202511591

**Published:** 2025-07-21

**Authors:** Niklas Geue, Marc Safferthal, Kevin Pagel

**Affiliations:** ^1^ Institute of Chemistry and Biochemistry Freie Universität Berlin Altensteinstraße 23a 14195 Berlin Germany; ^2^ Department of Molecular Physics Fritz‐Haber‐Institut der Max‐Planck‐Gesellschaft Faradayweg 4‐6 14195 Berlin Germany

**Keywords:** Collision‐induced dissociation, Gas‐phase mechanisms, Gas‐phase spectroscopy, Glycan fragmentation, Ion mobility mass spectrometry (IM‐MS)

## Abstract

Structural alterations in oligosaccharides are often associated with disease, positioning clinical glycomics as an emerging tool for diagnostics. This is most commonly achieved using a controlled collision‐induced dissociation (CID) of larger oligosaccharides into fragments and measuring their mass in a mass spectrometer. Due to the complexity of oligosaccharides, and particularly their unusual fragmentation mechanisms, the underlying processes are poorly understood. Deciphering glycan fragmentation and making it understandable is highly desirable and would transform the field of glycomics from an expert technique into a widely applicable tool available to non‐specialists. Here, we review the current knowledge of glycan fragmentation mechanisms in CID, with particular emphasis on hexose migrations and the anomeric memory. We discuss challenges and perspectives for future investigations, opening the window to widespread use of glycomics in clinical applications based on a fundamental understanding of glycan fragmentation.

## Graphical abstract

Glycan fragmentation with collision‐induced dissociation (CID) mass spectrometry (MS) leads to a range of specific disassembly mechanisms, which are being reviewed in detail.

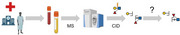

## 1 Introduction

**Figure 1:**
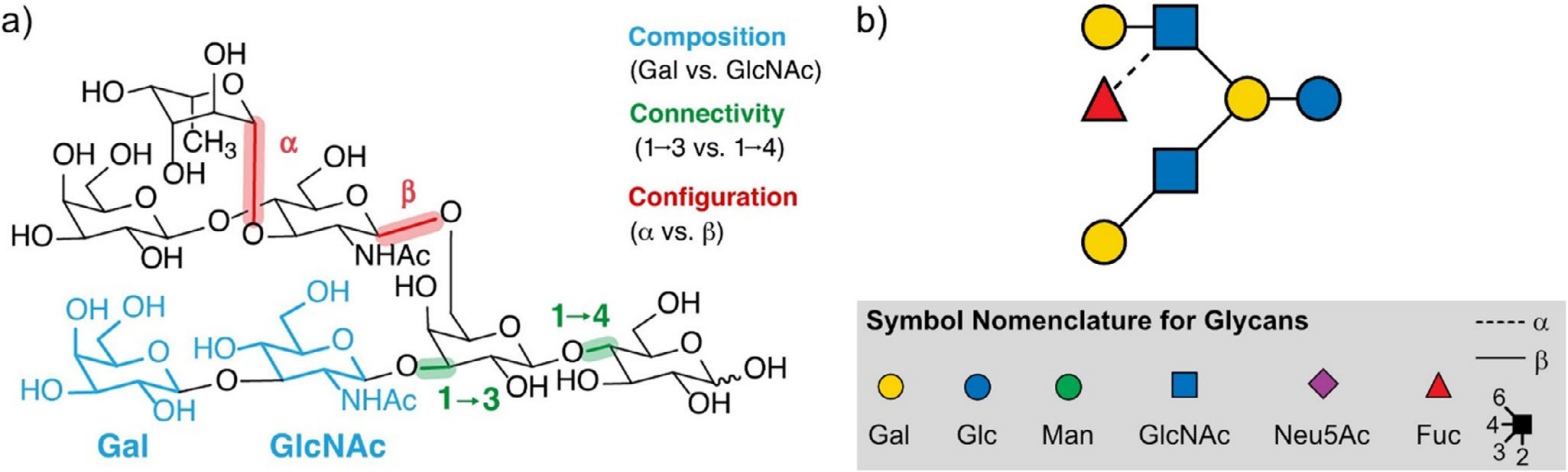
a) The structural complexity of glycans. Oligosaccharides differ in their monosaccharide building blocks (composition, blue), regiochemistry (connectivity, green), and stereochemistry (configuration, red), as highlighted in the exemplary structure. This complexity often results in isomers. b) Symbol nomenclature of glycans, including the monosaccharides displayed in this manuscript, as well as anomeric and linkage information. Adapted with permission from Ref. [9] © 2017 Elsevier Ltd.

Carbohydrates are ubiquitous in nature and represent the most abundant biopolymers found on the earth.^[^ ^1^ ^]^ In most living systems, shorter and structurally diverse sugar chains, often referred to as oligosaccharides or glycans, play a major role in the sensing of inter‐ and intramolecular interactions, such as in immune responses. Similar to proteins, the function of glycans is directly linked to their structure, which is often characterised by a distinct monosaccharide composition, regio‐ and stereochemistry (Figure 1).^[^ ^2^ ^]^ This complexity represents a major challenge in the analysis of glycans and is one of the reasons that glycans remain largely underexplored compared to proteins^[^ ^3^ ^]^ despite glycan composition being known to alter with disease. A prominent example is the correlation between mucin‐type *O*‐glycans and cystic fibrosis,^[^ ^1^, ^4^ ^]^ where people with cystic fibrosis show significantly altered sulfation and sialylation in their sputum glycans.^[^ ^5^ ^]^ Changes in the glycome are also associated with the occurrence of lung cancer,^[^ ^6^ ^]^ prostate cancer^[^ ^7^ ^]^ and ulcerative colitis,^[^ ^8^ ^]^ demonstrating the widespread potential of glycomics to target sugars for diagnostics and treatments.

The entire complement of glycans in an organism is referred to as the glycome, and their analysis (glycomics) — similarly to proteomics and genomics — is crucial in life sciences.^[^ ^10^, ^11^ ^]^ Typically, glycomics experiments rely on high‐throughput mass spectrometry (MS) techniques coupled to orthogonal separation techniques such as liquid chromatography, capillary electrophoresis or ion mobility spectrometry (IMS).^[^ ^12^, ^13^ ^]^ Particularly for larger glycans, or those attached to proteins (glycoproteins) and lipids (glycolipids), an unambiguous identification with the methods above is difficult, and the fragmentation of glycans in tandem mass spectrometry (MS^2^) experiments is often required to obtain meaningful information.^[^ ^14^, ^15^, ^16^ ^]^ The most used MS^2^ method is collision‐induced dissociation (CID), in which ions are activated through multiple collisions with gas atoms or molecules.^[^ ^17^ ^]^ Fragment structures and dissociation mechanisms are highly diverse and difficult to predict due to the mechanism of CID, which limits the information obtained from omics workflows. A fundamental understanding of the dissociation behaviour is hence highly desirable for clinical applications.

The efforts to unravel glycan fragmentation mechanisms are in many ways comparable to the studies on peptide fragmentation and sequence scrambling performed over the first two decades of this century.^[^ ^18^, ^19^ ^]^ Here, gas‐phase spectroscopy was crucial to fully unravel the molecular details of rearrangement and fragmentation processes occurring during the CID analysis of peptides.^[^ ^20^ ^]^ The obtained results made peptide fragmentation understandable and predictable, which was one of the major requirements for the advancement of proteomics. In comparison to peptides, glycan fragmentation is considerably more complex and difficult to predict. Currently, there are very few systematic studies on glycan fragmentation, hampering widespread application of MS^2^ based glycomics and glycoproteomics.^[^ ^14^ ^]^

In this minireview, we discuss recent approaches to unravel the characteristics of glycan fragmentation, including associated hexose and charge migration reactions, as well as anomeric, linkage and ring‐size memory upon fragmentation. With that, we not only summarise existing results but also aim to inspire further investigations in this area.

## 2 Collision‐Induced Dissociation

CID is the most established method to activate and dissociate ions, in particular biomolecular species such as glycans. As such, it is implemented in most commercial mass spectrometers. It is also referred to as collision‐activated dissociation (CAD), or when using higher energies, as higher‐energy collisional dissociation (HCD); however, these all follow the same principle and similar fragmentation reactions.

### 2.1 Mechanism

**Figure 2:**
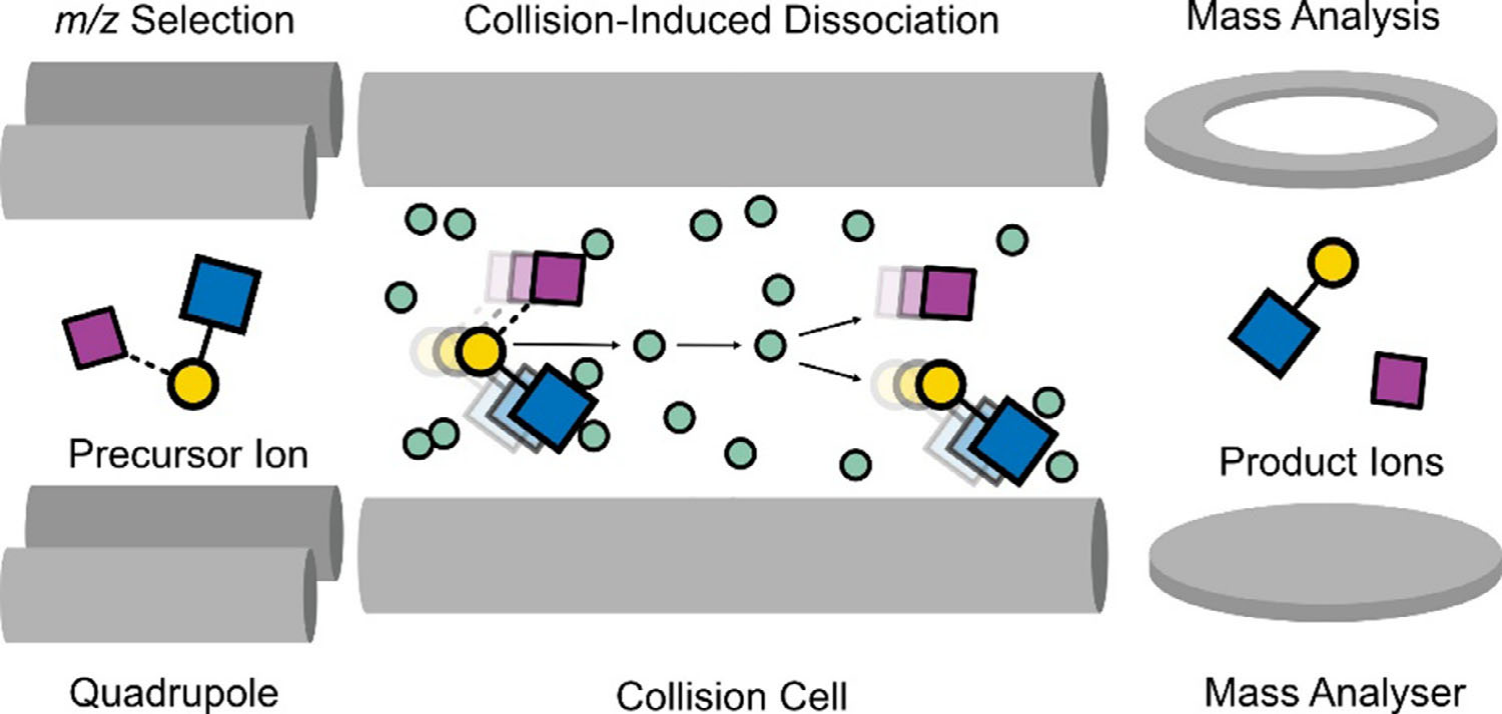
Fragmentation mechanism of CID on an example of a trisaccharide. Precursor ions are *m/z‐*selected in a quadrupole and accelerated in a collision cell. The ions collide multiple times with neutral gas, leading to an increase of internal energy and bond cleavage. The product ions contain structural information that can be analysed by MS.

In CID, ions are accelerated by an electric field into a cell filled with an inert gas (often Ar or N_2_), leading to inelastic collisions (Figure 2). The kinetic energy of the precursor ion depends on the potential difference of the electric field, and part of this energy is converted to internal energy upon collision, elevating the ion to an excited state.^[^ ^21^ ^]^ The internal energy from each collision is distributed among all internal nodes, eventually leading to nonspecific dissociation via the lowest energy fragmentation channels, which often involve the most labile bonds. This is the case, as the dissociation rate is low with respect to the rate of energy redistribution, making CID a slow ion activation method (on a micro‐ to millisecond time scale).^[^ ^14^, ^22^ ^]^ Notably, fragmentation can also proceed via higher energy pathways if sufficient energy is transferred. Commercial instrumentation involving CID often operates under multiple collision conditions, in which already excited ions are further excited by extensive collisions, slowly increasing the internal energy over time.^[^ ^22^ ^]^ This has the advantage of a more efficient energy transfer and higher product ion yields; however, it also leaves time for rearrangement prior to dissociation, which is an important topic of this minireview.^[^ ^21^, ^22^ ^]^ CID usually operates in the eV range, whereas in HCD, a commercial nomenclature of CID, energies of up to hundreds of eV can be applied.^[^ ^23^ ^]^ Their mechanisms are highly similar; however, HCD is in general faster and involves less collisions. It can also fragment heavier ions, for which energy in the eV range is not sufficient. HCD is usually not required for the fragmentation of pure glycans but can be relevant for the fragmentation of glycoconjugates such as glycopeptides or glycolipids, which have higher masses than isolated glycans and usually require more energy to fragment.^[^ ^24^ ^]^

### 2.2 Collision‐Induced Dissociation of Glycans

**Figure 3:**
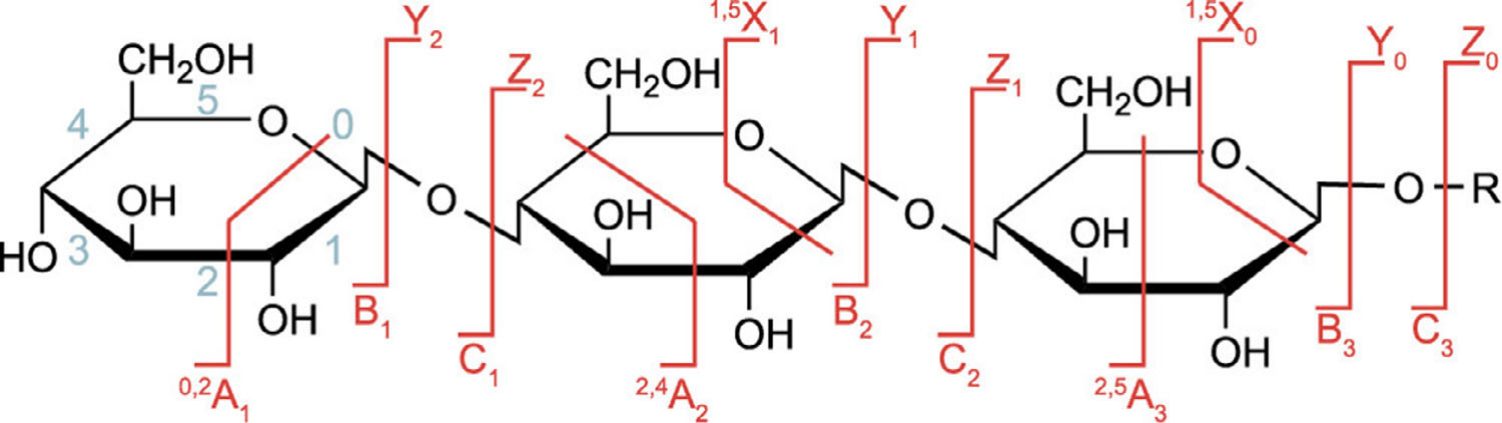
Nomenclature of glycan fragments according to Domon and Costello.^[^ ^25^ ^]^ A‐/X‐fragments are the result of cross‐ring cleavage, whereas B‐/Y‐ and C‐/Z‐fragments are caused by glycosidic cleavage (subscript: cleavage position, superscript: cross‐ring bonds broken in the monosaccharide unit). To distinguish the fragments from those of peptides, capital letters are used. Reproduced with permission from Ref. [14] Copyright © 2021 The Authors.

In general, two types of dissociation are found in the CID spectra of glycans: cross‐ring cleavage, where fragmentation breaks intact monosaccharide units (leading to A‐/X‐fragments), and glycosidic cleavage, where the fission event occurs between two glycan units at the glycosidic bond (leading to B‐/Y‐ and C‐/Z‐fragments, Figure 3).^[^ ^14^, ^25^ ^]^ A‐, B‐ and C‐fragments contain the nonreducing end, whereas X‐, Y‐ and Z‐ions are the counterparts with the reducing end.^[^ ^14^ ^]^ Starting at the reducing end with 0, the cleavage position is counted in subscript numerals, and superscript numbers indicate the bonds that are broken during cross‐ring cleavage, if applicable.^[^ ^14^ ^]^

Glycosidic cleavage fragments dominate the spectra and inform on glycan sequence; however, the structural knowledge obtained is limited as they provide little information about branching and the stereochemistry of the glycosidic bond at first glance.^[^ ^26^ ^]^ Cross‐ring cleavages are more informative, especially with respect to the connectivity and branching,^[^ ^26^, ^27^ ^]^ however, often multiple isomeric fragments can be formed and a clear interpretation of the spectra is difficult. The analysis of cross‐ring fragments is further complicated by their low abundance, which is partially because at least two bonds must be broken. In general, the cleavage of multiple bonds in glycan ions poses challenges for both glycosidic bond and cross‐ring cleavages, as it often leads to isomeric internal fragments, which can complicate sequence derivation.

In addition to the specific glycan fragmentation mechanisms discussed in the chapters below, the ion polarity in the mass spectrometer as well as the investigated adduct have a tremendous impact on the fragmentation mechanism. For example, negative ions generally show more cross‐ring cleavages than positive ions,^[^ ^28^, ^29^ ^]^ but even amongst ions of the same polarity there are considerable differences. Regardless of ion polarity and adduct, the underlying fragmentation mechanisms are poorly understood to date.^[^ ^14^ ^]^

Similar to peptides and proteins, differences in fragmentation channels are also found when comparing beam‐induced CID, for example, in quadrupole collision cells with CID in ion traps.^[^ ^30^ ^]^ In addition to the differences in energetics, which are in general difficult to compare across instruments and collision cell geometries, the nature of the fragment types seems to vary. This effect warrants further investigations and needs to be considered when comparing CID spectra of the same ion across instruments.

## 3 Mass Spectrometry‐Based Techniques for the Analysis of Glycan Fragments

The key for verifying glycan fragmentation pathways is orthogonal gas‐phase techniques, such as IMS and gas‐phase infrared (IR) spectroscopy. Both methods can be coupled with MS, enabling the characterisation of fragments generated by CID in the same instrument.

### 3.1 Ion Mobility Spectrometry (IMS)

**Figure 4:**
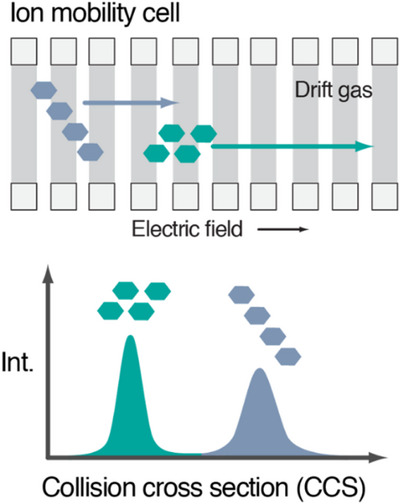
Principles of ion mobility separation. Ions with identical mass and composition can be separated based on their CCS, which enables the distinction of conformers and isomers. Reproduced with permission from Ref. [35] © 2021 The Authors.

IMS separates ions based on their time to traverse a gas‐filled cell under the guidance of an electric field. Larger and more extended structures exhibit more collisions with the inert gas than smaller, compact ions, leading to differences in the arrival times (Figure 4).^[^ ^22^, ^31^ ^]^ Hence, arrival times are diagnostic for the ions’ structures and can further be converted to instrument‐ and method‐independent rotationally averaged collision cross sections (CCS). These can be compared to literature or theoretical CCS values computed from candidate structures, often derived from quantum chemical methods such as density functional theory (DFT). This approach enables the direct structural assignment of ions with IMS. Recent advances in instrumentation have significantly improved IMS resolution, enabling the identification of glycan fragments^[^ ^32^ ^]^ and even the separation of glycan anomers.^[^ ^33^, ^34^ ^]^

### 3.2 Gas‐Phase Infrared Spectroscopy

**Figure 5:**
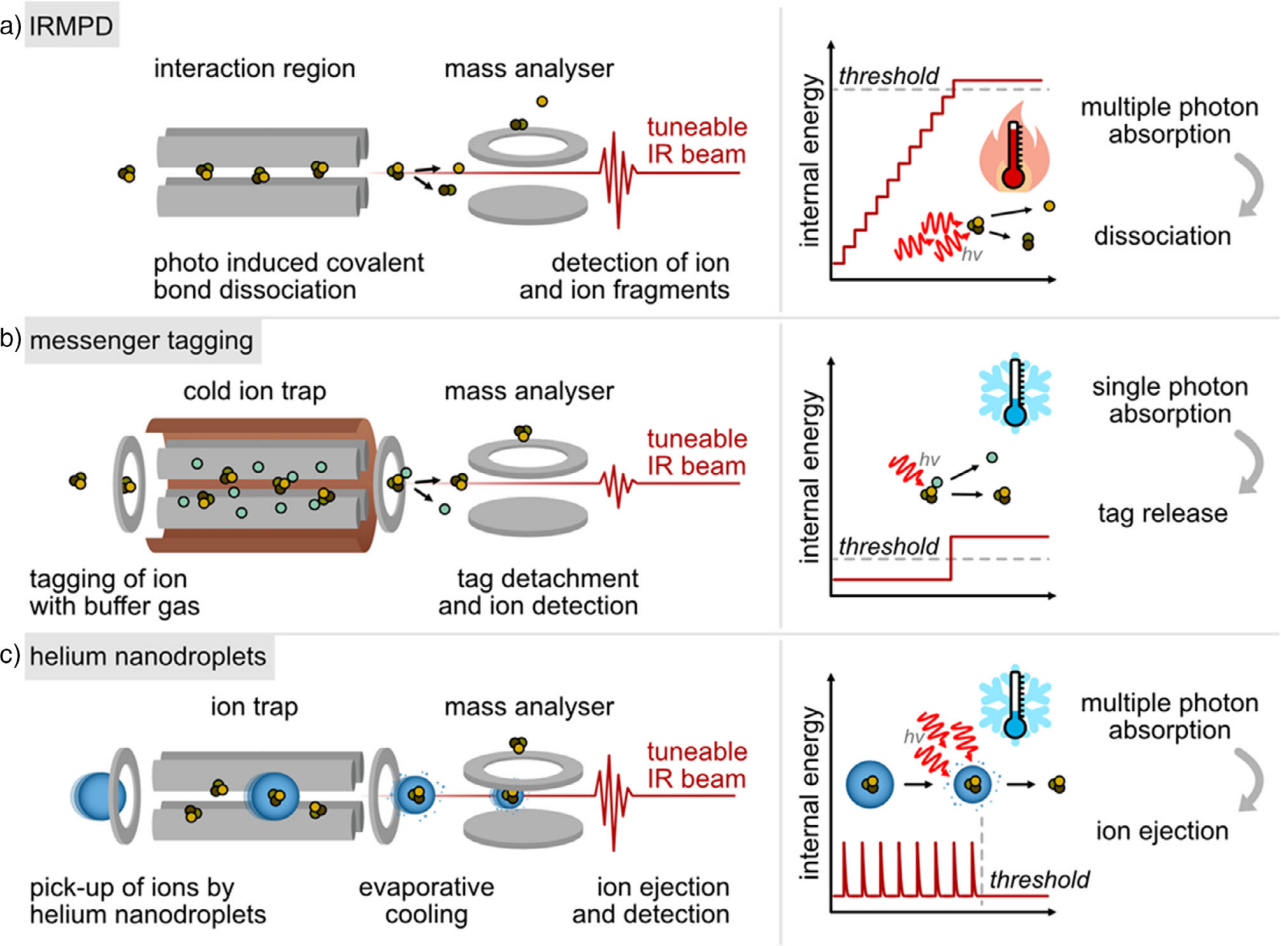
Illustration of a) infrared multiphoton dissociation (IRMPD), b) messenger tagging, and c) helium nanodroplet IR spectroscopy, including instrument setup (left) and spectroscopy principle (right). Adapted from Ref. [41] with permission from the Royal Society of Chemistry.

Another promising development in the field of glycan fragmentation analysis is structural identification by gas‐phase IR spectroscopy.^[^ ^36^, ^37^ ^]^ When IR radiation is in resonance with a vibrational transition of a fragment ion, the absorption of photons occurs and the ion becomes vibrationally excited. Due to the low particle density in gas‐phase experiments, the absorption of photons must be indirectly monitored through action spectroscopy, often measured by following the fragmentation of ions and subsequent detection of these fragments by MS. The problem with this is that the energy of IR photons is often lower than what is required for dissociation, and hence multiple photons need to be absorbed for the internal energy to be raised to the dissociation threshold. This method is known as infrared multiphoton dissociation (IRMPD, Figure 5a),

A common issue in gas‐phase IR spectroscopy is peak broadening that partly arises from the thermal activation of ions during the sequential absorption of multiple photons.^[^ ^38^ ^]^ The resulting spectral congestion can therefore limit IRMPD to smaller glycans and their fragments. This can; however, be addressed using method variants at cryogenic temperatures, such as messenger‐tagging IR spectroscopy and IR spectroscopy in helium nanodroplets.^[^ ^39^ ^]^ In messenger‐tagging spectroscopy (Figure 5b), ions are tagged with a buffer gas (often He, Ar or N_2_), and irradiation in a cryogenic ion trap at the resonant frequency leads to the release of the tag and the appearance of the untagged ion in the mass spectrum. As only one photon is absorbed, spectral broadening is reduced.

The third method presented here is gas‐phase IR spectroscopy with helium nanodroplets (Figure 5c), in which ions are picked up by ultracold helium droplets prior to spectroscopy. When irradiated with a resonant photon, the ion is excited and immediately cooled again to its ground state by evaporation of He from the droplet shell. After several absorptions, the ion is eventually released from the droplet and detected by MS. This approach has shown the highest resolving power so far; however, it is currently only available to expert groups.^[^ ^40^ ^]^ More details about gas‐phase IR spectroscopy techniques and their application for glycans can be found elsewhere.^[^ ^14^, ^39^ ^]^ Similarly to IMS, experimentally obtained gas‐phase IR spectra can be compared to the computed frequencies based on DFT calculations, yielding highly diagnostic fingerprints that often lead to unambiguous identification of glycan fragment structure.^[^ ^14^ ^]^

## 4 Hexose Migrations

The most prominent glycan‐specific fragmentation mechanisms are hexose migrations,^[^ ^42^ ^]^ which were first observed by McNeil in per‐*O*‐alkylated oligosaccharide alditols in 1983.^[^ ^43^ ^]^ In hexose migrations, an internal hexose residue dissociates from the glycan, migrates to a different position within the sequence and forms a new bond, likely on a sub‐microsecond timescale.^[^ ^44^ ^]^ This behaviour can often be easily monitored with CID, as an internal residue loss (IRL) frequently accompanies this hexose migration via the same mechanism.^[^ ^45^ ^]^ It can be detected through the fragment mass, which is different from the usual ordered sequencing of the glycan chain.^[^ ^42^ ^]^ As a consequence, IRL can lead to altered glycan sequences and misassignments in glycomics.^[^ ^42^ ^]^ Hence, understanding the driving force and mechanism of this phenomenon is important to apply glycan sequencing with confidence to clinical samples.

### 4.1 Influence of Adduct, Derivatisation and Reducing End

Hexose migrations occur almost exclusively in protonated ions or other adducts with mobile protons,^[^ ^46^ ^]^ as suggested by Wuhrer already in 2011. This statement seems to have held ever since, and glycomics experiments are recommended to avoid protonated ions.^[^ ^46^ ^]^ Alkali metal adducts show no hexose migration,^[^ ^42^ ^]^ and have all been proposed as measures to avoid migration rearrangements in glycomics, along with alkylammonium adducts,^[^ ^46^ ^]^ fluorescent markers,^[^ ^47^ ^]^ and free radicals.^[^ ^48^ ^]^ Deprotonated ions in negative mode are usually not prone to hexose migrations; however, Hsu and Turk observed rearrangements for negatively charged sulfatide ions in some cases.^[^ ^49^ ^]^

It was hypothesised that capping of free hydroxyl groups through peracetylation^[^ ^50^ ^]^ or permethylation^[^ ^43^ ^]^ might prevent rearrangements; however, this was not the case. Similarly to unprotected glycans, protonated species [M + H]^+^ undergo migration reactions readily, whereas it is not the case for sodiated ions.^[^ ^42^ ^]^

For glycoconjugates, particularly glycoproteins, MS^2^ is often realised with protonated ions, enabling the same migration reactions discussed here for glycans. As this field is beyond the scope of this minireview, we will not discuss it in much detail; however, it was found that the reducing end itself can potentially migrate.^[^ ^42^, ^51^ ^]^ Systematic investigations in this ‘reducing end migration’ are warranted.

### 4.2 Fucose Migration

The most prominent example of IRL is fucose migration, a term coined by Ernst et al. in 1997.^[^ ^52^ ^]^ Since then, several mechanisms have been proposed,^[^ ^42^, ^45^ ^]^ and the circumstances around which fucose migration occurs have been investigated in detail using ion mobility mass spectrometry (IM‐MS)^[^ ^53^ ^]^ and gas‐phase IR spectroscopy.^[^ ^46^, ^54^, ^55^, ^56^ ^]^ The main requirement for fucose migration is the presence of mobile protons, or, at least poor, charge fixation, most commonly found in protonated glycans [M + x H]^x+^.^[^ ^42^, ^46^ ^]^ Ammonium adducts are similarly prone to fucose migration, whereas alkylated ammonium adducts are not, as no mobile proton is present.^[^ ^46^ ^]^ Migration further seems to be independent of fucose linkage position and anomericity,^[^ ^57^ ^]^ but tends to occur more strongly when the residues are spatially close.^[^ ^42^, ^56^ ^]^ Notably, Franz and Lebrilla reported long‐range glycosyl transfer reactions as early as 2002, suggesting that the high collision environments in CID bring distant residues closer to each other.^[^ ^58^ ^]^ Fucose migration between different antennae of the same glycan ion was also found.^[^ ^44^, ^59^ ^]^

**Figure 6:**
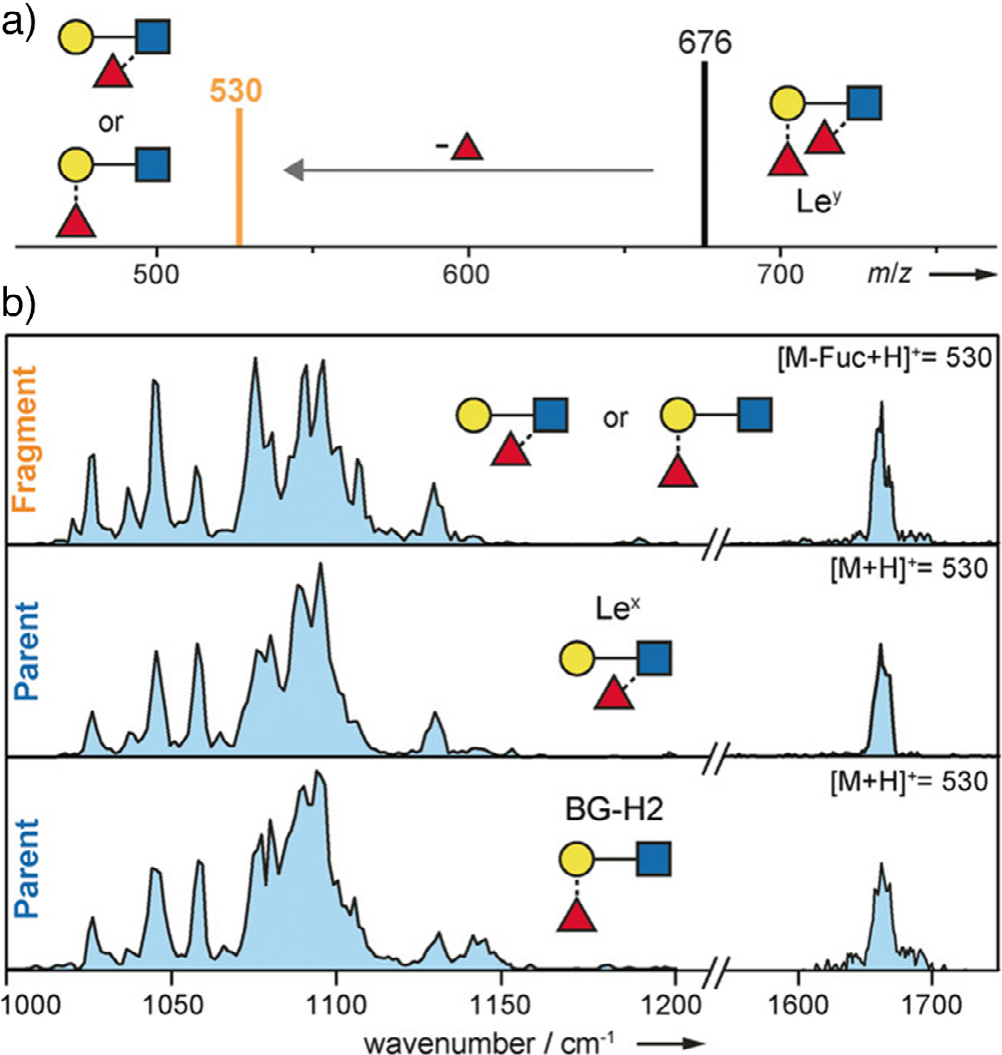
a) Schematic CID‐MS spectrum of the Le^y^ antigen as the [M + H]^+^ species. The main fragment (*m*/*z* = 530) is the result of fucose migration. b) Gas‐phase IR spectra of Le^y^ fragment and intact trisaccharide precursor standards Le^x^ and BG‐H2 (all *m*/*z* = 530). Reproduced from Ref. [54] © 2018 Wiley‐VCH Verlag GmbH & Co. KGaA, Weinheim.

Despite the progress made in this area, to date, the exact fucose migration mechanism and the molecular structure(s) of the rearrangement product are still debated. Mucha et al. used gas‐phase IR spectroscopy to show that fucose migration also occurs in intact protonated oligosaccharide ions in the absence of CID, suggesting that fucose migration is a universal phenomenon in MS due to a low energy barrier (Figure 6).^[^ ^54^ ^]^ The authors hypothesised that charge migration of the mobile proton catalyses the rearrangement of individual fucose residues to spatially adjacent sites. More recently, Moge et al. found an ‘intra‐residue fucose migration’, in which a fucose residue located at O3 or O4 of a GlcNAc residue migrates to O6 of the same residue.^[^ ^56^ ^]^ This is further evidence for a low energy barrier of fucose migration in the gas phase.

In 2023, both Lettow et al. and Moge et al. aimed to decode the fucose migration product in Lewis‐type antigens, yielding conflicting results.^[^ ^55^, ^56^ ^]^ Using a combination of IM‐MS, gas‐phase IR spectroscopy and DFT, Lettow et al. found an α(1–6)glycosidic bond to the galactose residue to be the most likely product, whereas Moge et al. concluded that binding occurs to the O6 of the *N*‐Acetylglucosamine.

As these data are not conclusive, further systematic studies are required to decipher the migration product, as well as the exact mechanism. For the latter, several hypotheses have been made; however, the results are not conclusive either. In oligosaccharides modified at the reducing end with 2‐aminobenzamide, it has been proposed that the nitrogen atom of the linker initiates a nucleophilic attack, facilitating the transfer of the migrating residue and subsequent cleavage of the terminal glycosidic bond.^[^ ^45^ ^]^ This mechanism combines a migration process that does not depend on the internal loss of residues, followed by fragmentation. An alternative reaction pathway has been suggested, wherein migration occurs to a distant hydroxyl group within the oligosaccharide.^[^ ^58^ ^]^ Other functional groups, aside from the amine linker, are also plausible candidates for migration destinations, as IRL is observed in oligosaccharides with methylated amine linkers. In glycans that involve sialic acids and GlcNAcs, it has been proposed that the oxygen atom of the amide group attacks the anomeric carbon of fucose, forming a new bond and leading to the generation of an imine group, with the proton located at the reducing end of the chain.^[^ ^52^ ^]^ A recent study by Kontodimas et al. suggests that the *N*‐acetyl groups in GlcNAc strongly influence the likelihood of fucose migrations depending on the glycan sequence.^[^ ^60^ ^]^ Migration is further facilitated when the amide group brings the mobile proton near the glycosidic bond of the fucose. When the mobile proton gets locked in a strong hydrogen bond between the amide and the oxygen atom in the ring, fucose migration is inhibited.

### 4.3 Migration of Other Monosaccharides

While fucose migration is the most prominent and investigated type of glycan rearrangement reaction, other building blocks may also migrate,^[^ ^42^ ^]^ including rhamnose,^[^ ^61^, ^62^ ^]^ glucuronic acid,^[^ ^61^ ^]^ and mannose,^[^ ^51^ ^]^ as well as the pentose xylose.^[^ ^61^, ^63^ ^]^ Xylose is so far the only non‐hexose unit observed to migrate; however, this does not exclude the occurrence of migration reactions for other monosaccharide units.

Haverkamp and co‐workers studied reducing and non‐reducing oligosaccharides and found a rearrangement of rhamnose residues — a deoxy sugar similar to fucose — in their protonated ions.^[^ ^61^ ^]^ Later, Ma et al. hypothesised a mechanism in which the ring oxygen is first protonated, leading to subsequent cleavage of the adjacent C1─O bond.^[^ ^62^ ^]^ This results in a carbenium ion at the anomeric stereocenter of the migrating group. At the end, the oxygen atom of the flavonoid residue at the reducing end of the diglycoside attacks the previously formed carbenium ion, and, as a result, an internal residue is eliminated from the oligosaccharide. More than a decade later, Wuhrer et al. discovered hexose migrations for non‐deoxy sugars, namely mannose residues in reductively aminated *N*‐glycans.^[^ ^51^ ^]^ Furthermore, Hecht et al. found β‐1,2‐xylose to migrate to glucosamine residues in *N*‐glycans in 2017.^[^ ^63^ ^]^ While different monosaccharide units have been found to migrate, it remains unclear how general this behaviour is and what factors influence the rearrangement reactions. Further studies, including those of a mechanistic nature with IMS and gas‐phase IR spectroscopy, are warranted to fully understand monosaccharide migrations in glycans.

## 5 Anomeric Memory

Glycans can occur in different configurations (α and β), resulting in two different anomers per glycosidic bond (Figure 1), and hence an exponentially growing number of diastereomers in longer oligo‐ and polysaccharides. As glycosidic cleavages are the dominant fragmentation channels in glycans, the question of whether and how the anomeric centre is retained upon CID is crucial for deciphering the sugar code. Glycosidic cleavages lead to two categories of fragment ions: B‐ and Z‐ions, which both do not contain the glycosidic oxygen anymore, as well as C‐ and Y‐ions, where the glycosidic oxygen is retained (Figure 3).

**Figure 7:**
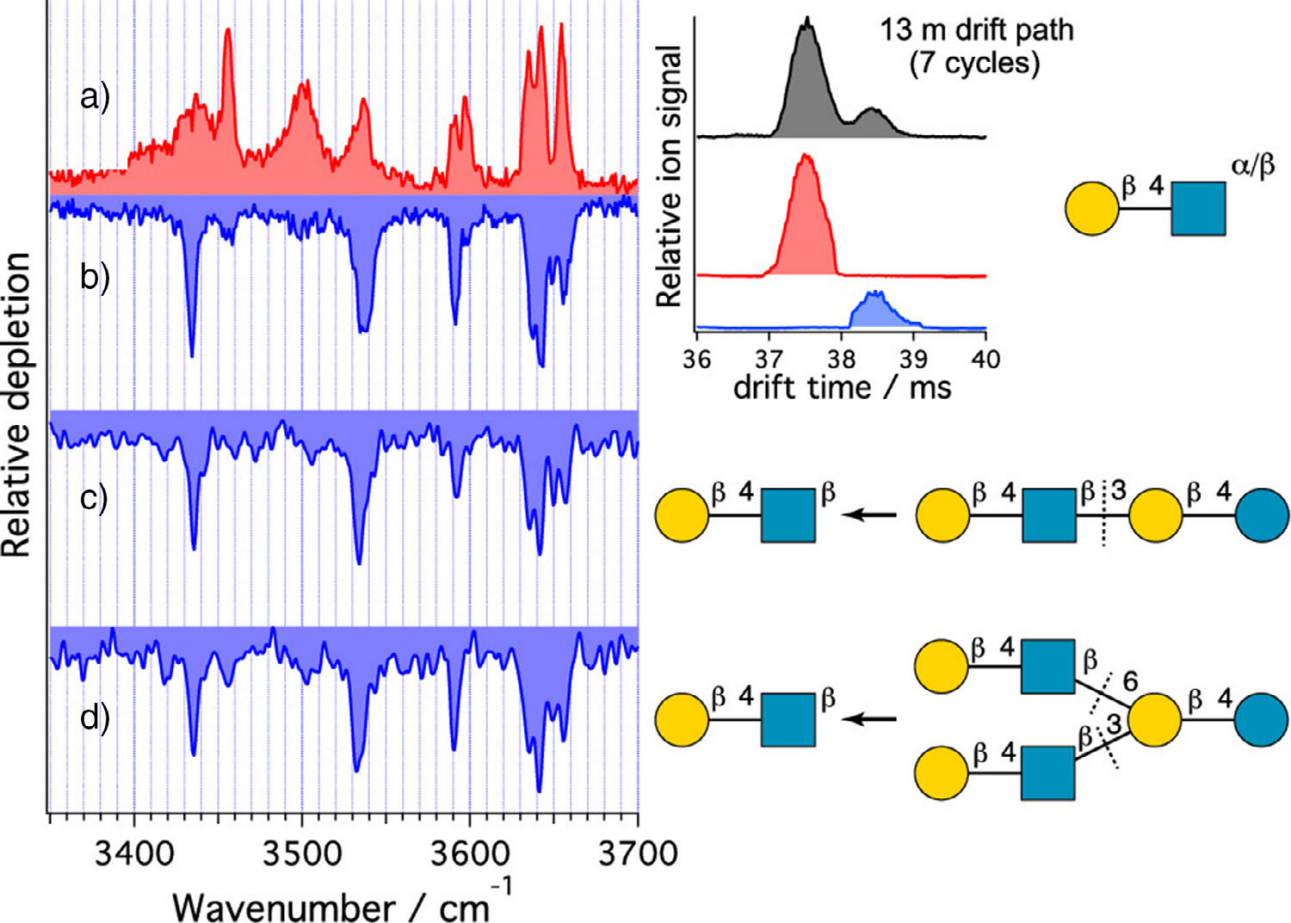
Comparison between the cryogenic IR spectra of the separated a) α‐anomer and b) β‐anomer of Galβ(1–4)GlcNAc and the C_2_ fragments from the human milk oligosaccharides c) LNnT and d) LNnH. Inset: high‐resolution arrival time distribution of Galβ(1–4)GlcNAc. Reproduced with permission from Ref. [66] Copyright © 2020 American Chemical Society.

In C‐ and Y‐ions, no bonds are broken at the anomeric centre, and hence an anomeric memory was hypothesised to be likely by Schindler et al.^[^ ^64^ ^]^ The first evidence of this effect was found by Gray et al., who used *^18^O*‐labelling, IM‐MS, gas‐phase IR spectroscopy and ab initio calculations to investigate the fragmentation of lithiated diglucoside standards and larger plant metabolite glycoconjugates.^[^ ^65^ ^]^ The resulting C‐fragments were unambiguously identified as closed‐ring α‐ or β‐glucose, maintaining the configuration of the precursor ion, respectively. Compagnon and co‐workers confirmed these findings for other C‐fragments in 2019,^[^ ^64^ ^]^ before Rizzo and co‐workers extended the trend for larger oligosaccharides (up to hexasaccharides) and for fragments larger than C_1_ (Figure 7 for the human milk oligosaccharides LNnT and LNnH).^[^ ^66^, ^67^ ^]^ Based on their results, they suggested that the anomeric memory of the C‐fragment is the rule rather than the exception, possibly due to the large mutarotation barrier in the gas phase. Ni and co‐workers found overall good anomeric memory for most C_2_‐ions by an alternative measurement route, involving the comparison of the C_2_‐ion CID spectra to databases of the respective disaccharide. Interestingly, no anomeric memory was found for some linkages, and the authors suggested a CID mechanism to linear C‐type ions as an explanation for the lack of anomeric memory.^[^ ^68^ ^]^ Ollivier et al. found that the anomeric centre was also retained in Y‐fragments of lithiated carbohydrates in multistage IMS experiments,^[^ ^69^ ^]^ which was also confirmed by Rizzo and co‐workers for sodiated oligosaccharides^[^ ^70^ ^]^ as well as Guttman and co‐workers for protonated disaccharides.^[^ ^71^ ^]^

There is some evidence for an anomeric memory in B‐ and Z‐type fragments, which is more surprising as the stereocenter vanishes completely when such a glycosyl cation is formed via CID. This could be explained through rearrangement reactions leading to different fragment structures. Though the details of the effect remain unclear,^[^ ^72^ ^]^ Rabus et al. suggested certain mechanisms for Z‐fragments of deprotonated glycans computationally.^[^ ^73^ ^]^ Experimentally, both Gray et al. and Ujma et al. found some indirect evidence for an anomeric memory in B‐type fragments of lithiated and protonated oligosaccharides, respectively; however, the data was not conclusive and both studies suggested isomerism (for example, of the charge carrier site) as an alternative explanation.^[^ ^34^, ^65^ ^]^ Later, Ollivier et al. found an anomeric memory of B‐type ions using IM‐MS, although it was less pronounced than for C‐ or Y‐ions.^[^ ^69^ ^]^ Interestingly, Greis et al. studied B‐type fragment ions of protected galactosides, and no anomeric memory occurred.^[^ ^72^ ^]^ The foundation of this effect was unclear and does not exclude an anomeric memory in unprotected glycans, as the hydroxy groups could play an important role as previously suggested.^[^ ^65^ ^]^ Taken together, there is evidence for an anomeric memory in all fragments based on glycosidic cleavage; however, more studies are required to evaluate whether this effect is general.

## 6 Other Fragmentation Specifics for Glycans

### 6.1 Ring‐Size Memory

Recently, Compagnon and co‐workers investigated the ring‐size memory of galactose‐containing oligosaccharides for the first time, showing that precursor ions that contain galactofuranose (five‐membered ring) and galactopyranose (six‐membered ring) retain the same ring size upon dissociation.^[^ ^74^ ^]^ This came as a surprise to the authors, considering the major rearrangements in the dissociation and the high flexibility of the furanose ring, particularly in B‐fragments.

### 6.2 Charge Migration

To the best of our knowledge, no study has so far directly reported charge migration upon fragmentation in CID; however, it seems likely that this phenomenon is a factor. Using a combination of IMS and molecular dynamics simulations, Struwe et al. reported the migration of the negative charge in deprotonated hexasaccharides solely upon transfer to the gas phase.^[^ ^75^ ^]^ In particular, the charge was suggested to be delocalised over various hydroxy groups, and this phenomenon would also explain the overall instability of negatively charged glycan ions in CID.^[^ ^28^ ^]^ As observed for fucose migration, effects that occur during the ionisation process in general also tend to be important upon fragmentation,^[^ ^54^ ^]^ and this suggests that charge migration is a factor in the fragmentation of deprotonated glycan ions.

### 6.3 Linkage Memory

In addition to the anomeric memory, glycan fragments can exhibit a linkage memory based on their connectivity (Figure 1). Using IM‐MS and hydrogen‐deuterium exchange MS, Mookherjee et al. found that the Y‐fragments of protonated disaccharides retained a memory of their precursor.^[^ ^71^ ^]^ This linkage memory was rationalised with different bridging situations and ring rearrangements; however, direct evidence and a systematic understanding of this effect are currently lacking. Later, Ni and co‐workers found that cross‐ring cleavages of sodiated oligosaccharides can inform on the linkage position of the sugar at the reducing end.^[^ ^68^ ^]^ This cross‐ring dissociation is based on the retro‐aldol reaction, and differences in the resulting A‐ and X‐fragment intensity can be correlated with precursor linking, likely due to different intensities of secondary fragmentation.

## 7 Relevance of Glycan Fragmentation Mechanisms for Clinical Glycomics

Glycomics is already an essential tool for the quality control of glycosylated biopharmaceuticals such as monoclonal antibodies. First studies demonstrate its use for the early detection of congenital diseases and the mechanisms involved in host‐pathogen interactions.^[^ ^76^ ^]^ Significant changes in the glycome have also been found as possible progressive markers of disease in cancers, showing great potential in early stage diagnosis. For example, a decrease in galactosylation of IgG_1_ Fc *N*‐glycans was observed in lung cancer.^[^ ^6^ ^]^ Downregulation of high‐mannose and upregulation of sialylated tri‐ and tetraantennary *N*‐glycans, as well as changes in the sialic acid linkages, were determined as diagnostic markers for epithelial ovarian cancer.^[^ ^77^, ^78^ ^]^ An increase of α(2–3)‐sialic acid and a decrease of core fucosylation of prostate‐specific antigen *N*‐glycans can help to improve prostate cancer diagnosis.^[^ ^7^ ^]^

To observe these subtle differences in biopharmaceuticals or changes in the glycome, glycan fragments generated by CID are the most convenient and diagnostic source of information to date. Universal fragmentation mechanisms such as mannose and fucose migration currently inhibit the correct structural assignment in glycan profiling and are circumvented in routine analysis using negative ion mode fragmentation or fluorescent labels trapping protons in positive ion mode. A complete picture of glycan fragmentation mechanisms, similar to those of peptides for proteomics,^[^ ^18^, ^19^ ^]^ would simplify and accelerate glycan assignments rapidly and democratise glycomics workflows. Particularly important for top‐down glycomic workflows is an understanding of whether structural features are retained upon fragmentation, as discussed here for anomericity, linkage and ring‐size.

**Figure 8:**
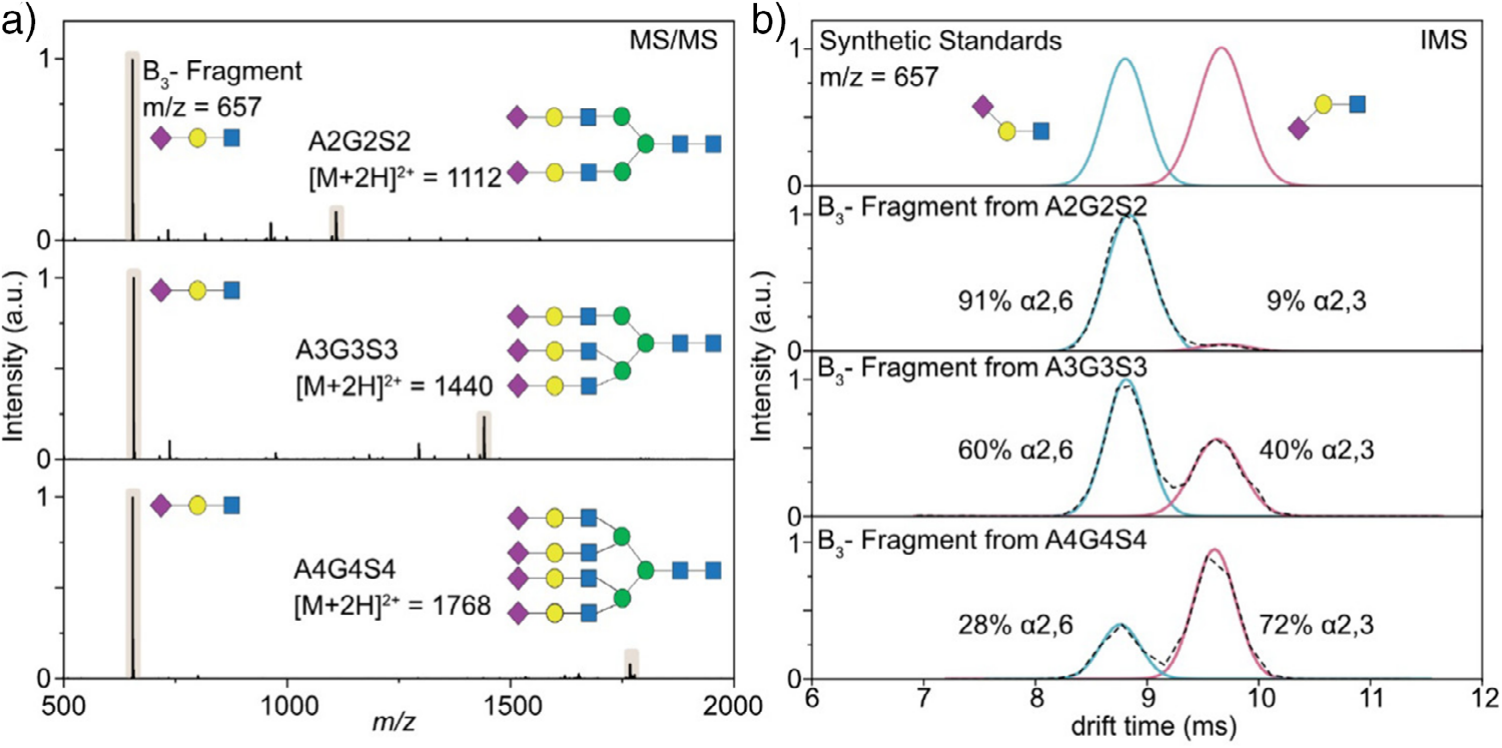
Determination of sialic acid isomers by IMS of B_3_ fragments from *N*‐glycans. a) MS^2^ spectra of bi‐, tri‐ and tetraantennary *N*‐glycans generated using CID. b) Comparison of mobilograms of two synthetic standards and B_3_ fragments from three sialylated *N*‐glycans. Adapted with permission from Ref. [79] Copyright © 2022 The Authors.

An interesting example, which demonstrates the connection of the glycan fragmentation mechanism with glycomics, is the differentiation between α(2–3) and α(2–6) sialic acid linkages commonly located at the terminal end galactose of *N*‐ and *O*‐glycans. This information plays a crucial role, as sialic acids can function as receptors for the hemagglutinin (HA) protein involved in influenza virus infections, and binding of HA with glycans only occurs when the preferred linkage type is present. Due to the rapid loss of sialic acids in traditional CID experiments, assessment of the sialic acid linkage is extremely challenging. Multiple studies have shown that the sialic acid linkage is retained in B_3_ trisaccharide fragments, which are easily generated in positive ion mode. Manz et al. generated the B_3_ fragments from sialylated *N*‐glycans by CID and separated the isomeric fragments by IMS.^[^ ^79^ ^]^ Comparison with reference standards ensured the assignment of the fragments, and the extent of α(2–3) and α(2–6) sialylation was quantified using IMS (Figure 8).

Sialylated glycans are commonly decorated by *O*‐acetyl groups, which can play an important role in host‐pathogen interactions. These labile modifications rapidly migrate in the solution phase between different hydroxyl groups of the same sialic acid. To assess the stability of *O*‐acetyl groups in MS experiments, Yeni et al. performed CID and gas‐phase IR spectroscopy on *O*‐acetylated GlcNAcs.^[^ ^80^ ^]^ Surprisingly, no sign of *O*‐acetyl group migration or losses was observed on the protonated molecular ions in the gas‐phase IR experiments. More recently, Vos et al. applied this idea in a combined MS^2^‐IM‐MS approach, showing that *O*‐acetyl isomers can be distinguished and assigned by screening for B_1_‐monosaccharide and B_3_‐trisaccharide fragments in *N*‐ and *O*‐glycans.^[^ ^81^ ^]^ Even though the field of clinical glycomics is still in its infancy, these two examples showcase that mechanistic fragmentation studies and structural elucidation of fragments will be central to the advancement of glycomics in the future.

## 8 Summary and Outlook

Despite being highly complex, glycan fragmentation mechanisms in CID are worth studying due to their relevance in clinical glycomics. Hexose migrations complicate the analysis of glycan fragment spectra significantly, whereas other effects such as the anomeric memory suggest that top‐down glycomics is a viable tool for the future of disease diagnostics. In contrast to peptides,^[^ ^18^, ^19^, ^20^ ^]^ the mechanisms of glycan fragmentation are still poorly understood, and we hope that this minireview will inspire further and more systematic investigations into the different disassembly pathways. This would not only open the window for a more widespread use of glycomics but also benefit the increasingly important analysis of intact glycoproteins and glycolipids (where the oligosaccharide backbone usually fragments at lower collision energies than the backbone structure).^[^ ^14^ ^]^ We predict that the development of instrumentation, such as the inclusion of IMS and cryogenic gas‐phase IR spectroscopy in one instrument,^[^ ^82^ ^]^ as well as the advancement of artificial intelligence will benefit the elucidation of glycan fragmentation mechanisms.^[^ ^83^ ^]^

## Conflict of Interests

The authors declare no conflict of interest.

## Data Availability

Data sharing is not applicable to this article as no new data were created or analysed in this study.
